# *Chrysanthemum indicum* Prevents Hydrogen Peroxide-Induced Neurotoxicity by Activating the TrkB/Akt Signaling Pathway in Hippocampal Neuronal Cells

**DOI:** 10.3390/nu13113690

**Published:** 2021-10-20

**Authors:** Yun Hee Jeong, Tae In Kim, You-Chang Oh, Jin Yeul Ma

**Affiliations:** Korean Medicine (KM)-Application Center, Korea Institute of Oriental Medicine, 70 Cheomdanro, Dong-gu, Daegu 41062, Korea; runxi0333@kiom.re.kr (Y.H.J.); tikim@kiom.re.kr (T.I.K.)

**Keywords:** *Chrysanthemum indicum*, neuroprotective effects, antioxidant, nuclear factor erythroid 2-related factor 2, tropomyosin-related kinase receptor B, protein kinase B

## Abstract

Oxidative stress-mediated neuronal damage is associated with the pathogenesis and development of neurodegenerative diseases. *Chrysanthemum indicum* has antioxidant properties. However, the neuroprotective effects and the cellular mechanism of *C. indicum* ethanol extract (CIE) against oxidative damage in hippocampal neuronal cells have not been clearly elucidated. Therefore, this study investigated whether CIE has protective effects against hydrogen peroxide (H_2_O_2_)-induced oxidative toxicity in HT22 cells. CIE pretreatment significantly improved neuronal cell viability. Moreover, the formation of intracellular reactive oxygen species and apoptotic bodies, and mitochondrial depolarization were significantly reduced in HT22 cells with H_2_O_2_-induced oxidative toxicity. Furthermore, CIE increased the phosphorylation of tropomyosin-related kinase receptor B (TrkB), protein kinase B (Akt), cAMP response element-binding protein, the expression of brain-derived neurotrophic factor, antioxidant enzymes, and the nuclear translocation of nuclear factor erythroid 2-related factor 2 by activating the TrkB/Akt signaling pathway. In contrast, the addition of K252a, a TrkB inhibitor, or MK-2206, an Akt-selective inhibitor, reduced the neuroprotective and antioxidant effects of CIE. Taken together; CIE exhibits neuroprotective and antioxidant effects against oxidative damage. Therefore, it can be a potential agent for treating oxidative stress-related neurodegenerative diseases.

## 1. Introduction

Oxidative stress is a major cause of several neurodegenerative diseases including Alzheimer’s and Parkinson’s disease and cerebral ischemia [1,2,3]. In particular, central nervous system and neuronal cells, with high polyunsaturated fatty acid content and a high oxygen consumption rate, are susceptible to oxidative stress [4,5]. Reactive oxygen species (ROS) are important neuronal signaling molecules in normal physiological processes, including intracellular signal transduction and gene expression. However, the overproduction and accumulation of ROS, such as hydrogen peroxide (H_2_O_2_) and superoxide anion, can cause extensive oxidative stress responses, which, in turn, lead to mitochondrial dysfunction, cell damage, and death [6,7]. Therefore, reducing the risk of oxidative stress caused by the overproduction of ROS is an important target for treating or preventing neurodegenerative diseases.

Brain-derived neurotrophic factor (BDNF) plays an important role in several brain functions including neuronal survival, proliferation, protection, and synaptic plasticity [8,9,10]. Previous studies have shown that the expression of BDNF protects neuronal cells against oxidative stress and decreases the risk of neurodegenerative disorders in the brain [11,12]. BDNF elicits its physiological function by binding with the specific cell surface tropomyosin-related kinase receptor B (TrkB) receptor. Subsequently, the activation of BDNF/TrkB signaling leads to the phosphorylation and activation of the protein kinase B (Akt) and transcription factor cAMP response element-binding protein (CREB) [13,14]. Nuclear factor erythroid 2-related factor 2 (Nrf-2) is correlated with another mechanism of neuroprotection. It is a central transcription factor that can activate antioxidant enzymes within the central nervous system. The enzymes include heme oxygenase (HO)-1, NAD(P)H quinone oxidoreductase 1 (NQO1), glutamate–cysteine ligase catalytic subunit (GCLC), and glutathione (GSH) [15,16,17]. Several studies have shown that the Nrf-2-mediated pathways exhibit neuroprotective effects by directly reducing oxidative stress and maintaining the integrity of the mitochondria [18,19]. Therefore, the regulation of the TrkB/Akt/CREB/BDNF pathway and the Akt/Nrf-2/antioxidant enzyme in the brain may be a key point for the treatment or prevention of neurodegenerative diseases.

*Chrysanthemum indicum* (CI) is a medicinal plant found in East Asia. Further, it has been used traditionally to treat various conditions, such as inflammation, hypertension, and respiratory diseases in Korea, China, and Japan [20]. Previous studies reported that CI is beneficial as it has antibacterial, anti-inflammatory, immunomodulatory, antioxidant, and anticancer properties [21,22,23,24]. However, the inhibitory effects of *C. indicum* ethanol extract (CIE) against H_2_O_2_-induced neurotoxicity have not been elucidated. Therefore, the current study aimed to investigate the neuroprotective effects of CIE against H_2_O_2_-induced apoptosis via the modulation of the TrkB/Akt/CREB/BDNF pathway and the Akt/Nrf-2/antioxidant enzyme in HT22 cells. Moreover, the chemical components of CIE were evaluated via high-performance liquid chromatography (HPLC) analysis.

## 2. Materials and Methods

### 2.1. Materials and Reagents

Dulbecco’s Modified Eagle Medium (DMEM), fetal bovine serum (FBS), and antibiotics were obtained from Hyclone (Logan, UT, USA); dimethyl sulfoxide and H_2_O_2_ from Sigma-Aldrich (St. Louis, MO, USA); and a cell counting kit (CCK) from Dojindo Molecular Technologies, Inc. (Kumamoto, Japan). Moreover, 2′,7′-dichlorodihydrofluorescein diacetate (H_2_DCFDA) was acquired from Invitrogen (Carlsbad, CA, USA) and the Annexin V-FITC/propidium iodide (PI) apoptosis detection kit from BD Biosciences (Franklin Lakes, NJ, USA). Chloride salt JC-1 was obtained from Biotium (Hayward, CA, USA). Bradford reagent was purchased from Bio-Rad (Hercules, CA, USA). The polyvinylidene difluoride (PVDF) membrane was obtained from Millipore (Bedford, MA, USA). Primary antibodies and horseradish peroxidase (HRP)-conjugated secondary antibodies were purchased from Cell Signaling Technology, Inc. (Boston, MA, USA). Chlorogenic acid, luteoloside, and 3,5-dicaffeoylquinic acid were purchased from Sigma-Aldrich. Acetonitrile, a solvent for HPLC analysis, was obtained from Merck (Darmstadt, Germany), and phosphoric acid was provided by Sigma-Aldrich. Tertiary distilled water was prepared using Puris-Evo RO Water System (Mirae ST Co., Ltd., Anyang-si, Korea). All solutions (standard compound, distilled water, and 0.3% phosphoric acid mixed with water) were filtered before injection for analysis.

### 2.2. Preparation of CIE

The dried whole parts of CI were obtained from Yeongcheonhyundai Herbal Market (Yeongcheon, Korea) and were deposited in the herbal bank of KM-Application Center, Korea Institute of Oriental Medicine, after they were assessed by Prof. KiHwan Bae (College of Pharmacy, Chungnam National University, Korea). To prepare the CIE, the pulverized CI (30.0 g) was extracted with 390 mL of 70% ethanol at 40 °C in a shaking incubator (100 rpm) for 24 h. The extract solution was filtered using a 150-mm filter paper (Whatman, Piscataway, NJ, USA) and was concentrated using a rotary vacuum evaporator (Buchi, Tokyo, Japan). Samples were freeze-dried and kept in a desiccator at −20 °C before use.

### 2.3. Cell Culture

HT22 cells, which are mouse hippocampal neuronal cell lines, were cultured in DMEM supplemented with 10% FBS and 1% antibiotics (v/v). Cells were maintained at 37 °C in a saturated humidity atmosphere containing 5% CO_2_.

### 2.4. Cell Viability Assay

HT22 cells (5 × 10^3^ cells/well) were seeded and cultured into a 96-well plate for 24 h. The cells were pretreated with CIE at concentrations of 50, 100, or 200 μg/mL for 2 h and with H_2_O_2_ (500 μM) for 24 h. In total, 10 μL of CCK solutions was added to each well, and the cells were incubated for another 1 h. The absorbance was evaluated using a microplate reader (SpectraMax i3, Molecular Devices, San Jose, CA, USA) at 450 nm.

### 2.5. Lactate Dehydrogenase Assay

The release of lactate dehydrogenase (LDH) in the medium was evaluated using the LDH assay kit, according to the manufacturer’s instruction. After treatment with 500 μM H_2_O_2_ for 24 h, the cells were centrifuged, and 10 μL culture medium was transferred to a 96-well plate. The reaction solution (10 μL/well) was applied to each well, and the LDH activity was investigated using a microplate reader at 450 nm. The LDH release results were expressed as percentage of the control.

### 2.6. Intracellular ROS Determination

Intracellular ROS was detected with an ROS detection kit, according to the manufacturer’s instruction. After CIE and H_2_O_2_ treatments, the cells were washed twice in 1× assay buffer and were then incubated with 20 μM of H_2_DCFDA for 30 min at 37 °C in the dark. The distribution of DCF fluorescence was analyzed using a fluorescence microplate reader at an excitation wavelength of 488 nm and an emission wavelength of 535 nm. The representative fluorescence images were obtained with a fluorescence microscope (Eclipse Ti, Nikon, Tokyo, Japan).

### 2.7. Flow Cytometry Analysis

The presence of apoptotic cells was determined using the Annexin V-FITC/PI apoptosis detection kit. The cells were harvested 24 h after exposure to H_2_O_2_, washed with ice-cold Ca^2+^-free phosphate-buffered saline, and resuspended in binding buffer (0.1 M HEPES/NaOH, pH 7.4, 1.4 M NaCl, and 25 mM CaCl_2_). Next, 5 μL of Annexin V-FITC and 5 μL of PI were added and incubated for 15 min at room temperature (RT) in the dark. The cells were analyzed via flow cytometry (FACSCalibur, Becton Dickinson, CA, USA).

### 2.8. JC-1 Staining

JC-1, a fluorescent probe, was used to detect the mitochondrial membrane potential, according to the manufacturer’s instruction. Briefly, JC-1 working solution (5 mg/mL) was added to the medium for 15 min at 37 °C in the dark and was rinsed with culture media. Fluorescence images were obtained using a fluorescence microscope. The red (polymeric) fluorescence was visualized under an excitation wavelength of 520 nm and an emission wavelength of 590 nm. The green (monomeric) fluorescence was visualized under an excitation wavelength of 490 nm and an emission wavelength of 530 nm.

### 2.9. Western Blot Analysis

For Western blot analysis, the total cellular proteins were lysed using radioimmunoprecipitation assay lysis buffer (Millipore) by adding protease and phosphatase inhibitor cocktail (Roche, Basel, Switzerland). The protein concentration was determined using the Bradford method. Similar amounts of total protein were separated via sodium dodecyl sulfate–polyacrylamide gel electrophoresis and were electrically transferred onto the PVDF membranes. The membranes were blocked with 3% BSA at RT for 1 h and were incubated with their respective primary antibodies (1:1000) overnight at 4 °C. After washing, they were incubated with HRP-conjugated secondary antibodies for 1 h at RT. The relative intensity of the protein expression was quantitated using ChemiDoc^TM^ Touch Imaging System (Bio-Rad). The relative protein expression was determined using Image J software (version 1.53k) with normalization to the control value.

### 2.10. Nuclear and Cytosolic Protein Extraction

The cytosolic and nuclear fractions of HT22 cells were isolated with NE-PER Nuclear and Cytoplasmic Extraction Reagents (Thermo Fisher, Rockford, IL, USA), according to the manufacturer’s instruction.

### 2.11. Preparation of Extract Sample and Standard Solutions for HPLC Analysis

To prepare the extract sample, 30 mg of CIE was weighed and dissolved in 1 mL of methanol, extracted via ultrasonication, and filtered using the 0.2-μm PETE membrane syringe filter. After filtration, the filtrate was injected for HPLC analysis. For CIE analysis, chlorogenic acid, luteoloside, and 3,5-dicaffeoylquinic acid, which are standard compounds, were purchased and prepared at 1000 ppm (1 mg/mL) concentration using HPLC grade methanol with an ultrasonicator for 30 min. A calibration curve was prepared to determine the content of each standard component in CIE. The calibration curve was manufactured by preparing a high concentration of dilution stock and by diluting with methanol according to the concentration of each standard compound.

### 2.12. HPLC-DAD System

To identify the content of chlorogenic acid, luteoloside, and 3,5-dicaffeoylquinic acid in CIE, HPLC analysis was conducted using Dionex UltiMate 3000 (Dionex Corp., Sunnyvale, CA, USA), which is comprised of a binary pump, auto sampler, column oven, and diode array UV/VIS detector (DAD). Data processing, output signaling, and data analysis were processed with Dionex Chromeleon (Thermo Fisher).

### 2.13. HPLC Analysis Condition

To identify the content of chlorogenic acid, luteoloside, and 3,5-dicaffeoylquinic acid in CIE, HPLC analysis was performed with X bridge C18 Column (250 mm × 4.6 mm, 5 μm) connected with a C18 guard column cartridge. The eluted solvent was set up with A (0.3% phosphoric acid) and B (acetonitrile), and the flow rate was 1.0 min/mL. The analysis solvent was eluted with the gradient method: 0–25 min, 15–40% A; 25–35 min, 40–100% B. The chromatogram signal of CIE with its standard components was recorded according to HPLC condition: UV detection, 280 nm; column oven temperature, 30 °C; and injection volume, 10 μL. The limits of detection (LOD) and limits of quantification (LOQ) were calculated via calibration, which were prepared by injecting at each concentration. The LOD and LOQ values were calculated as 3.3 × σ/S and 10 × σ/S, respectively (where σ is the standard deviation of the noise from the regression equation and S is the slope of the calibration curve). The signal peak area was analyzed using chromatography software (Chromeleon 7), and all samples were injected in triplicate under the same environment.

### 2.14. Statistical Analysis

All results were expressed as means ± standard error of the mean, and the statistical significance was analyzed with GraphPad Prism version 5.02 (GraphPad Software, Inc., San Diego, CA, USA). Statistical significance was via one-way analysis of variance, followed by the Tukey’s test, after comparing each sample. The *p* values less than 0.05 were considered significant. Different alphabetical letters indicate statistically significant difference between values.

## 3. Results

### 3.1. Effects of CIE on H_2_O_2_-Induced Neurotoxicity in HT22 Cells

**Figure 1:**
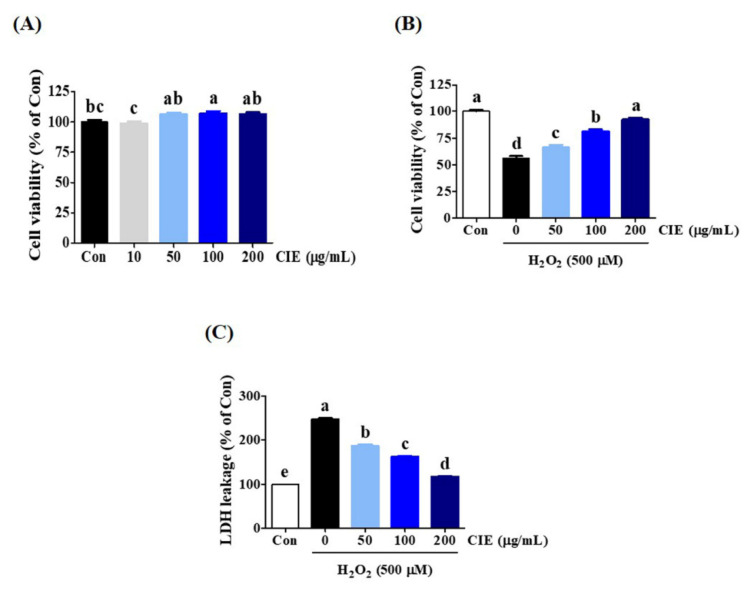
Effects of *Chrysanthemum indicum* ethanol extract (CIE) on hydrogen peroxide (H_2_O_2_)-induced cytotoxicity in HT22 cells. (**A**) HT22 cells were incubated with CIE at concentrations of 50, 100, and 200 μg/mL. (**B**,**C**) After CIE pretreatment at concentrations of 50, 100, and 200 μg/mL, HT22 cells were stimulated with H_2_O_2_ (500 μM). Control cells were incubated with the vehicle alone. Data are presented as mean ± standard error of the mean of the results of the three independent experiments. Con, control; LDH, lactate dehydrogenase. Different alphabetical letters on the bars (a–e) indicate statistically significant difference from each other (*p* < 0.05).

We first assessed the effect of CIE on the viability of HT22 cells using the CCK assay. Results showed that CIE treatment up to a concentration of 200 μg/mL alone for 24 h did not show cytotoxicity and had a slight proliferative effect (Figure 1A). Thus, subsequent experiments were performed with concentrations of <200 μg/mL. Next, to explore the possible protective effects of CIE against H_2_O_2_-treated neurotoxicity in HT22 cells, CCK and LDH assays were conducted. The HT22 cells were treated with CIE at concentrations of 50, 100, or 200 μg/mL for 2 h before exposure to 500 μM H_2_O_2_ for 24 h. As shown in Figure 1B, the cell viability decreased to approximately 50% in H_2_O_2_-treated cells alone. In contrast, CIE treatment at concentrations of 50, 100, and 200 μg/mL significantly improved cell viability, which was reduced by H_2_O_2_, in a concentration-dependent manner. Meanwhile, H_2_O_2_ treatment increased LDH leakage in H_2_O_2_-treated cells compared with control cells. Moreover, CIE pretreatment significantly decreased H_2_O_2_-induced LDH leakage (Figure 1C). Hence, CIE could remarkably prevent H_2_O_2_-induced neurotoxicity in HT22 cells.

### 3.2. Effects of CIE on H_2_O_2_-Induced Intracellular ROS Accumulation

**Figure 2:**
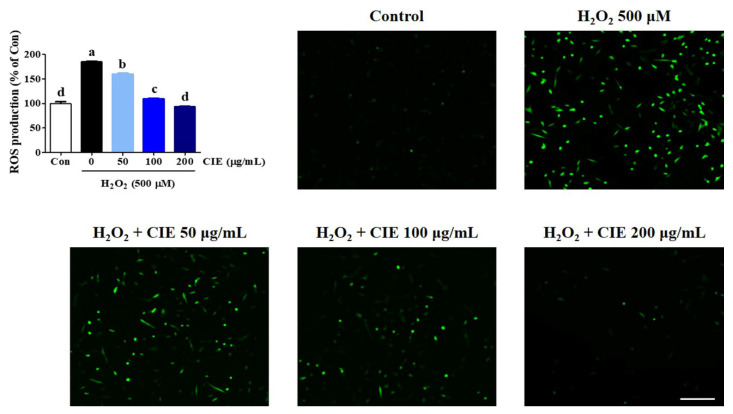
Effects of *Chrysanthemum indicum* ethanol extract (CIE) against hydrogen peroxide (H_2_O_2_)-induced intracellular reactive oxygen species (ROS) production in HT22 cells. Cells were pretreated with CIE at concentrations of 50, 100, and 200 μg/mL and then with 500 μM H_2_O_2_. H_2_DCFDA (20 μM), an oxidation-sensitive fluorescence dye, was used to assess ROS levels. The expression of ROS was determined using a fluorescence microscope and fluorescence microplate reader. Scale bar = 100 μm. Control cells were incubated with the vehicle alone. All experiments were repeated at least three times, and similar results were obtained. Data are presented as mean ± standard error of the mean. Con, control. Different alphabetical letters on the bars (a–d) indicate statistically significant difference from each other (*p* < 0.05).

The overproduction of ROS can cause oxidative stress, neuronal dysfunction, and cell death. Thus, we explored the level of ROS production in HT22 cells using a H_2_DCFDA fluorescence assay. As shown in Figure 2, the intracellular ROS level significantly increased after treatment with 500 μM H_2_O_2_ in H_2_O_2_-treated cells compared with control cells. Meanwhile, CIE pretreatment remarkably inhibited H_2_O_2_-induced ROS generation in a concentration-dependent manner. Staining with H_2_DCFDA was performed to confirm the expression of ROS level using a fluorescent microscope, and the results were similar to those mentioned above (Figure 2). Therefore, CIE can reduce the overproduction of ROS induced by H_2_O_2_.

### 3.3. CIE Restored Mitochondrial Membrane Potential Reduction of HT22 Cells Treated with H_2_O_2_

**Figure 3:**
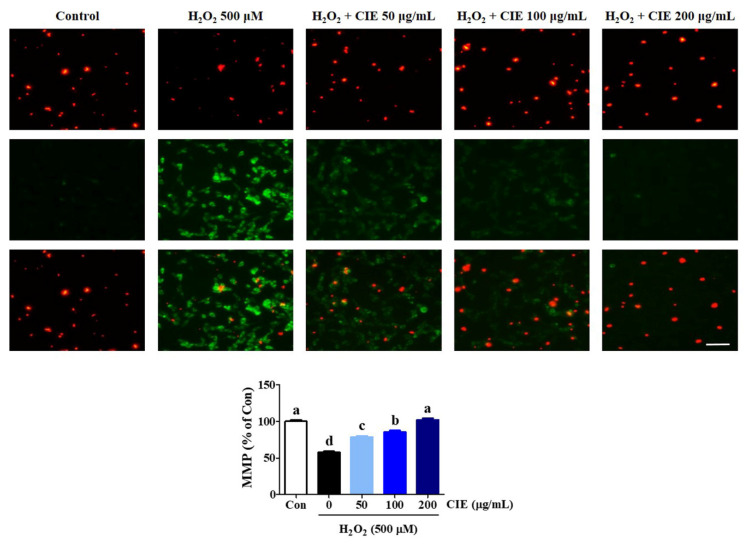
Effects of *Chrysanthemum indicum* ethanol extract (CIE) on hydrogen peroxide (H_2_O_2_)-induced mitochondrial dysfunction in HT22 cells. The mitochondrial membrane potential (MMP) was assessed via microscopy using JC-1 staining. Images are representative of the three independent experiments at magnification of 400×. Scale bar = 50 μm. Red fluorescence indicated normal MMP; green fluorescence, damaged mitochondria with MMP loss. The histogram shows the red/green fluorescence intensity ratio. Control cells were incubated with the vehicle alone. Data are presented as mean ± standard error of the mean of the results of the three independent experiments. Con, control. Different alphabetical letters on the bars (a–d) indicate statistically significant difference from each other (*p* < 0.05).

To examine whether CIE modulated mitochondrial function in H_2_O_2_-treated cells, we assessed MMP using JC-1 fluorescence staining assay. JC-1 exists as an aggregated form (red fluorescence) in the matrix of the healthy mitochondria. Meanwhile, the monomeric form was represented by green fluorescence when the mitochondria were depolarized during apoptotic cell death. As shown in Figure 3, the control group exhibited high red fluorescence and low green fluorescence. However, when cells were treated with H_2_O_2,_ the mitochondrial membrane rapidly changed. The green fluorescence intensity significantly increased, whereas the red fluorescence intensity significantly decreased, indicating MMP loss. In contrast, CIE pretreatment prevented MMP loss induced by H_2_O_2_, as evidenced by decreasing green fluorescence and increasing red fluorescence. In particular, CIE at a concentration of 200 μg/mL resulted in a JC-1 distribution similar to that in control cells. Hence, CIE had strong protective effects against MMP loss caused by H_2_O_2_-induced oxidative stress in HT22 cells.

### 3.4. CIE Inhibited Apoptotic Cell Death Induced by H_2_O_2_ in HT22 Cells

**Figure 4:**
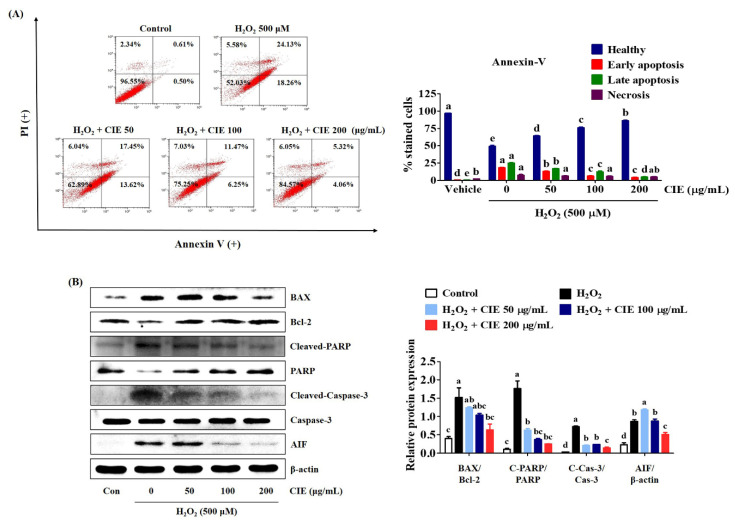
Effects of *Chrysanthemum indicum* ethanol extract (CIE) against hydrogen peroxide (H_2_O_2_)-induced apoptosis in HT22 cells. Cells were pretreated with CIE at concentrations of 50, 100, and 200 μg/mL and were then exposed to H_2_O_2_ (500 μM). (**A**) Apoptosis of HT22 cells was evaluated via flow cytometry. Quantitative data showed the percentage of healthy, early apoptotic, late apoptotic, and necrotic cells according to treatment. (**B**) The expression levels of BAX, Bcl-2, cleaved-PARP, cleaved-caspase-3, and AIF were determined via Western blot analysis. Control cells were incubated with the vehicle alone. Blot images were representative of the three independent experiments and data are presented as mean ± standard error of the mean. PI, propidium iodide; Con, control. Different alphabetical letters on the bars (a–e) indicate statistically significant difference from each other (*p* < 0.05).

To identify the protective effects of CIE on H_2_O_2_-induced cell death in HT22 cells, the apoptotic rates were examined via flow cytometry. After H_2_O_2_ treatment for 24 h, the percentage of apoptotic cells increased to about 50% (Figure 4A). However, CIE pretreatment significantly reduced H_2_O_2_-exposed cell death in apoptotic cell populations (Figure 4A). We further assessed the expression of apoptosis-related proteins including B-cell lymphoma 2 (Bcl-2), Bcl-2-associated X (BAX), cleaved-poly (ADP-ribose) polymerase (PARP), cleaved-caspase-3, and apoptosis-inducing factor (AIF) via Western blot analysis. As shown in Figure 4B, H_2_O_2_ treatment increased the production of apoptotic markers in HT22 cells. However, CIE pretreatment blocked the expression of BAX, cleaved-PARP, cleaved-caspases-3, and AIF. Moreover, it restored the level of anti-apoptotic factors, such as Bcl-2 and PARP, in CIE-treated cells compared with H_2_O_2_-treated cells. Thus, CIE had a neuroprotective effect via the reduction of H_2_O_2_-induced apoptotic cell death.

### 3.5. CIE Enhances the Expression of Antioxidant Enzymes via the Akt/Nrf-2 Signaling Pathway

**Figure 5:**
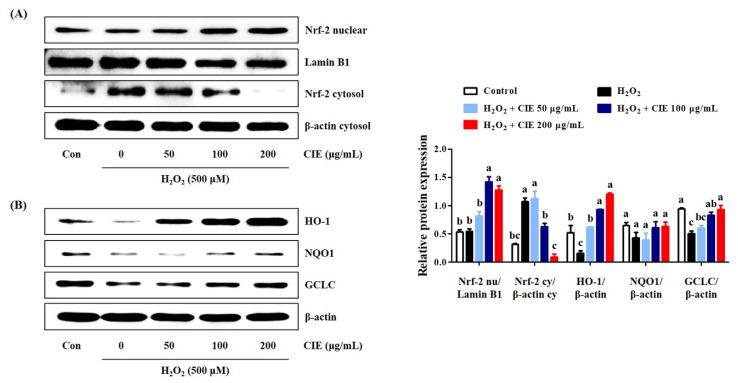
Effects of *Chrysanthemum indicum* ethanol extract (CIE) on the activation of Nrf-2 and antioxidant enzymes in hydrogen peroxide (H_2_O_2_)-exposed HT22 cells. Cells were incubated with 500 μM H_2_O_2_ with or without CIE. The expression levels of (**A**) nuclear and cytosolic Nrf-2, and (**B**) HO-1, NQO1, and GCLC were determined via Western blot analysis. Control cells were incubated with the vehicle alone. Blot images were representative of the three independent experiments and data are presented as mean ± standard error of the mean. Con, control. Different alphabetical letters on the bars (a–c) indicate statistically significant difference from each other (*p* < 0.05).

The activation of the Akt/Nrf-2/antioxidant response element (ARE) signaling pathway has been associated with neuroprotective effects [14]. Thus, we assessed whether CIE improved the expression of antioxidant enzymes, such as HO-1, NQO1, and GCLC via the regulation of the Akt/Nrf-2 signaling pathway. CIE pretreatment translocated cytosolic Nrf-2 to the nucleus (Figure 5A). Moreover, it effectively upregulated the expression levels of antioxidant enzymes including HO-1 and GCLC in H_2_O_2_-exposed HT22 cells (Figure 5B). However, under the same conditions, NQO1 expression was not significantly changed by H_2_O_2_ or CIE treatment. Therefore, CIE promoted the expression of antioxidant enzymes by regulating the Akt/Nrf-2 signaling pathway.

### 3.6. CIE Enhances Mature BDNF Expression via the Activation of the TrkB/Akt/CREB Pathway

**Figure 6:**
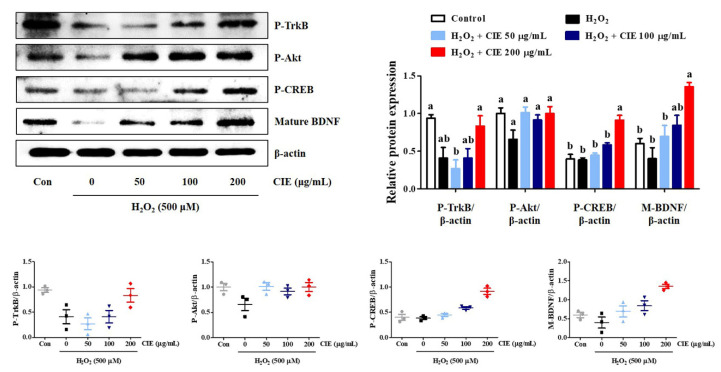
Effects of *Chrysanthemum indicum* ethanol extract (CIE) on the phosphorylation of TrkB, Akt, and CREB, and the expression of BDNF in hydrogen peroxide (H_2_O_2_)-exposed HT22 cells. Cells were pretreated with CIE at concentrations of 50, 100, and 200 μg/mL and then exposed to H_2_O_2_ (500 μM). Control cells were incubated with the vehicle alone. Blot images are representative of the three independent experiments and data are expressed as mean ± standard error of the mean. Con, control. Different alphabetical letters on the bars (a,b) indicate statistically significant difference from each other (*p* < 0.05).

The TrkB/Akt/CREB/BDNF signaling pathway is involved in neuronal growth and survival. To investigate the molecular mechanism underlying the neurotrophic action of CIE, Western blot analysis was conducted. As shown in Figure 6, CIE treatment slightly enhanced the phosphorylation of TrkB and Akt compared to H_2_O_2_-treated cells, but not significantly. However, 200 μg/mL CIE treatment significantly enhanced the phosphorylation of CREB and the expression of mature BDNF. Thus, CIE exhibited neuroprotective effects by activating the TrkB/Akt/CREB/BDNF signaling pathway, and in particular, more effectively induced the activation of CREB/BDNF.

### 3.7. K252a and MK-2206 Suppress the Neuroprotective Effect of CIE

**Figure 7:**
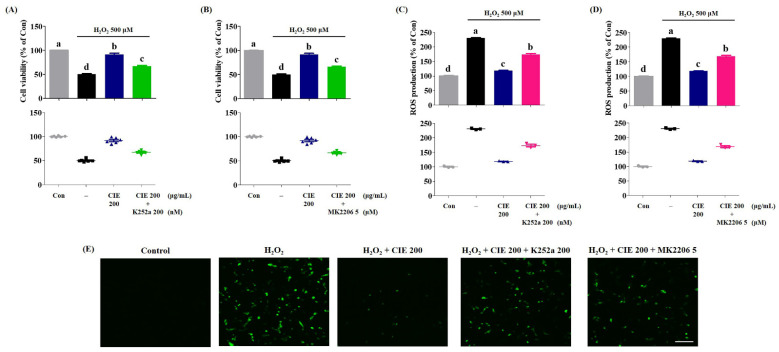
Suppressive effects of K252a or MK2206 on the neuroprotective action of *Chrysanthemum indicum* ethanol extract (CIE). HT22 cells were incubated with or without CIE combined with K252a or MK2206 before hydrogen peroxide (H_2_O_2_) treatment. (**A**,**B**) The cell viability and (**C**–**E**) the reactive oxygen species (ROS) level were assessed using CCK and H_2_DCFDA, respectively. Scale bar = 100 μm. Control cells were incubated with the vehicle alone. Data are presented as mean ± standard error of the mean of the results of the three independent experiments. Con, control. Different alphabetical letters on the bars (a–d) indicate statistically significant difference from each other (*p* < 0.05).

Subsequently, we confirmed the molecular mechanism of CIE that promotes the activation of the TrkB/Akt/CREB/BDNF and Akt/Nrf-2/ARE pathways in H_2_O_2_-treated cells. To examine the action of CIE, we evaluated the inhibitory activities of K252a, a TrkB inhibitor, and MK-2206, an Akt-selective inhibitor, in H_2_O_2_-treated cells. As shown in Figure 7, the inclusion of K252a or MK2206 combined with CIE effectively reduced the neuroprotective activity of CIE in H_2_O_2_-induced cell death. Further, the inhibitory mechanism reversed the suppressive effects of CIE on H_2_O_2_-induced ROS generation in HT22 cells. Thus, CIE prevented oxidative stress in hippocampal neuronal cells by promoting the expression of BDNF and antioxidant enzymes via the activation of the TrkB/Akt/CREB/BDNF and Akt/Nrf-2/ARE pathways.

### 3.8. Identification and Quantitative Analysis of the Chemical Constituents of CIE

**Figure 8:**
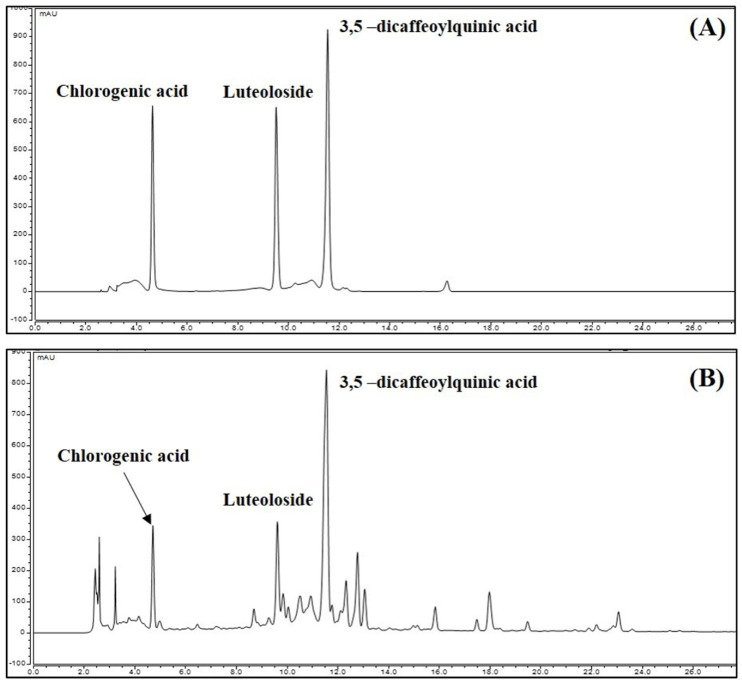
High-performance liquid chromatography chromatogram of (**A**) standard mixture and (**B**) *Chrysanthemum indicum* ethanol extract at 280 nm. Data showed each peak for chlorogenic acid (4.71 min), luteoloside (9.61 min), and 3,5-dicaffeoylquinic acid (11.55 min), respectively.

We performed an HPLC-DAD analysis to identify the contents of chlorogenic acid, luteoloside, and 3,5-dicaffeoylquinic acid in CIE. The standard constituents were detected selectively on the HPLC chromatogram at 4.710, 9.613, and 11.550 min. To the identified marker compound in CIE, we compared the UV spectrum (Supplementary Figure S1). The result was consistent with that of a previous study [25] (Figure 8). The calibration curves of the three compounds were y = 0.1661x − 0.8279, y = 0.2053x + 0.1165, and y = 0.2247x + 1.264, with coefficients of determination of 0.9996, 0.9995, and 0.9987, at the injected concentration ranges of 50.0–500.0 μg/mL (chlorogenic acid and luteoloside) and 75.0–750.0 μg/mL (3,5-dicaffeoylquinic acid), respectively (Table 1). Therefore, the calibration curve of the three compounds had good linearity at the tested concentration range. The contents of the three compounds were identified, and their area mean value in CIE was calculated using each constituent of the calibration curve equation. Based on the analysis results, the contents of chlorogenic acid, luteoloside, and 3,5-dicaffeoylquinic acid were 0.63%, 0.55%, and 2.13%, respectively.

## 4. Discussion

Oxidative stress caused by ROS plays an important role in the development of various neurodegenerative diseases including Alzheimer’s disease, Parkinson’s disease, and cerebral ischemia [1,2,3]. The overproduction and accumulation of ROS ultimately cause a series of oxidative stress responses, which, in turn, lead to mitochondrial dysfunction, cell damage, and death [6,7]. Therefore, a lower ROS generation may be useful for preventing and treating neurodegenerative diseases. CI is a medicinal plant that has been used traditionally as a therapeutic agent for various immune-related disorders in East Asia. Moreover, it has various physiological activities including antibacterial, anti-inflammatory, immunomodulatory, antioxidant, and anticancer properties [21,22,23,24]. Nevertheless, the efficacy and detailed mechanisms of CI against oxidative damage-mediated neurotoxicity in neuronal cells remain unknown. Thus, the current study investigated the neuroprotective effects and the potential mechanisms of CIE on H_2_O_2_-induced oxidative stress in HT22 cells.

Results showed that CIE pretreatment remarkably reduced H_2_O_2_-induced neuronal cell death, as evidenced by a high cell viability and low LDH release. In addition, CIE significantly inhibited the overproduction of ROS with H_2_O_2_ treatment in HT22 cells in a concentration-dependent manner. Mitochondrial membrane lipids respond very sensitively to the accumulation of ROS, resulting in dysfunction caused by MMP reduction [26]. This research showed that CIE treatment improves MMP reduction via H_2_O_2_ exposure in a concentration-dependent manner. Further, CIE inhibits the activation of mitochondrial apoptotic factors, including BAX, cleaved-PARP, cleaved-caspase-3, and AIF, thereby increasing the expression of anti-apoptotic factors, including Bcl-2 and PARP. Based on flow cytometry, CIE was effective in reducing the production of several apoptotic cell bodies. Hence, CIE pretreatment promotes neuronal cell survival by reducing mitochondrial apoptosis, thereby exerting a protective effect against H_2_O_2_-induced neurotoxicity.

BDNF, which is a neurotrophic factor, affects cell proliferation, differentiation, and synaptic plasticity by binding to the TrK receptor [12,27]. Therefore, BDNF is a crucial factor for the survival of neuronal cells exposed to oxidative stress. TrkB stimulated by BDNF induces the phosphorylation of Akt [28], and activated Akt can induce the secretion of BDNF by activating CREB in neuronal cells [29]. Therefore, whether CIE has neuroprotective effects by promoting the formation of BDNF by activating the TrkB/Akt/CREB signaling pathway was investigated. Western blot analysis revealed that CIE not only recovered the expression of BDNF, but also slightly increased TrkB and Akt phosphorylation and significantly increased CREB in HT22 cells exposed to H_2_O_2_. In contrast, K252a, a TrkB inhibitor, nullified the effects of CIE on cell viability and ROS production in H_2_O_2_-treated cells. Therefore, CIE could protect neuronal cells against oxidative stress-induced apoptosis by promoting the production of BDNF via the activation of the TrkB/Akt/CREB signaling pathway in HT22 cells.

The other possible mechanism for the neuroprotective effect of CIE may be correlated with the regulation of antioxidant enzymes, such as HO-1, NQO1, and GCLC, by activating the Akt/Nrf-2/ARE signaling pathway in neuronal cells. In the unstimulated state, Nrf- 2 is present in the cytoplasm in an inactive form bound to kelch-like ECH-associated protein 1 (KEAP1). Nrf-2 released from KEAP1 via oxidative stress stimulation, or the activation of Akt that translocates into the nucleus and binds to the ARE, promotes the expression of antioxidant enzymes in neuronal cells [30]. In addition, the activation of TrkB by BDNF facilitates the activation of AKT, which translocates cytoplasmic Nrf-2 to the nucleus, thereby enhancing the activity of antioxidant enzymes. Our experimental results showed that CIE pretreatment significantly increased Nrf-2 nuclear translocation and the production of antioxidant enzymes such as HO-1 and GCLC in CIE-treated cells compared with H_2_O_2_-treated cells. Moreover, CIE slightly upregulated the P-TrkB and P- Akt levels. In contrast, MK-2206, an Akt-selective inhibitor, and K252a, a TrkB inhibitor inhibited the neuroprotective activity of CIE in H_2_O_2_-treated cells. Therefore, CIE may have neuroprotective effects via the activation of the TrkB/Akt/Nrf-2 signaling pathway.

To determine the chemical components of CIE, we performed phytochemical analyses using HPLC. We identified three main components, which were chlorogenic acid, luteoloside, and 3,5-dicaffeoylquinic acid. A previous study showed that chlorogenic acid has antioxidant, anti-inflammatory, antibacterial, and free radical scavenging effects. Moreover, it enhances immunomodulatory ability [31,32,33,34,35]. Similarly, luteoloside (luteolin-7-O-glucoside; cymaroside) has anti-inflammatory, free radical scavenging, antibacterial, and antitumor properties [36,37,38]. Further, 3,5-dicaffeoylquinic acid has inhibitory effects against oxidative stress, inflammation, and gene mutations [39,40,41]. The current HPLC analysis and previous studies revealed that the neuroprotective effects of CIE may be closely correlated with the activity of chlorogenic acid, luteoloside, and 3,5-dicaffeoylquinic acid.

## 5. Conclusions

CIE pretreatment had neuroprotective effects against oxidative stress-induced cell apoptosis in HT22 cells. CIE was effective in suppressing H_2_O_2_-induced neurocytotoxicity and LDH leakage and in significantly reducing ROS overproduction and mitochondrial dysfunction. In addition, it significantly decreased the levels of BAX, cleaved-PARP, cleaved-caspase-3 and AIF and increased the expression of Bcl-2 and PARP. Furthermore, CIE pretreatment can protect neuronal cells against H_2_O_2_-induced neurotoxicity via the activation of the TrkB/Akt/Nrf-2 signaling pathway in HT22 cells. Therefore, CIE can be a potential agent for preventing and treating neurodegenerative diseases.

## Figures and Tables

**Table 1:** Calibration curves of compounds.

Compound	Range (μg/mL, ppm)	Regression Equation	*r* ^2^	LOD (μg/mL)	LOQ (μg/mL)
**1**	50.0~500.0	y = 0.1661x − 0.8279	0.9996	0.0080	0.0242
**2**	50.0~500.0	y = 0.2053x + 0.1165	0.9995	0.0064	0.0196
**3**	75.0~750.0	y = 0.2247x + 1.2640	0.9987	0.0059	0.0179

Chlorogenic acid (**1**); Luteoloside (**2**); 3,5-Dicaffeoylquinic acid (**3**). LOD = 3.3 × σ/*S*. LOQ = 10 × σ/*S*. σ is the standard deviation of the intercept from the regression equation and *S* is the slope of the calibration curve.

## Data Availability

The data presented in this study are available on request from the corresponding author.

## Supplementary Materials

The following are available online at www.mdpi.com/2072-6643/13/11/3690/s1, Figure S1. UV chromatogram of each standard compounds and CIE.
