# Supplementary figures and images for: Chrysanthemum indicum Prevents Hydrogen Peroxide-Induced Neurotoxicity by Activating the TrkB/Akt Signaling Pathway in Hippocampal Neuronal Cells
Source: Nutrients. 2021 Oct 20;13(11):3690. doi: 10.3390/nu13113690 (PMC8618340; doi:10.3390/nu13113690)

Supplementary data

Figure S1. UV chromatogram of each standard compounds and CIE.

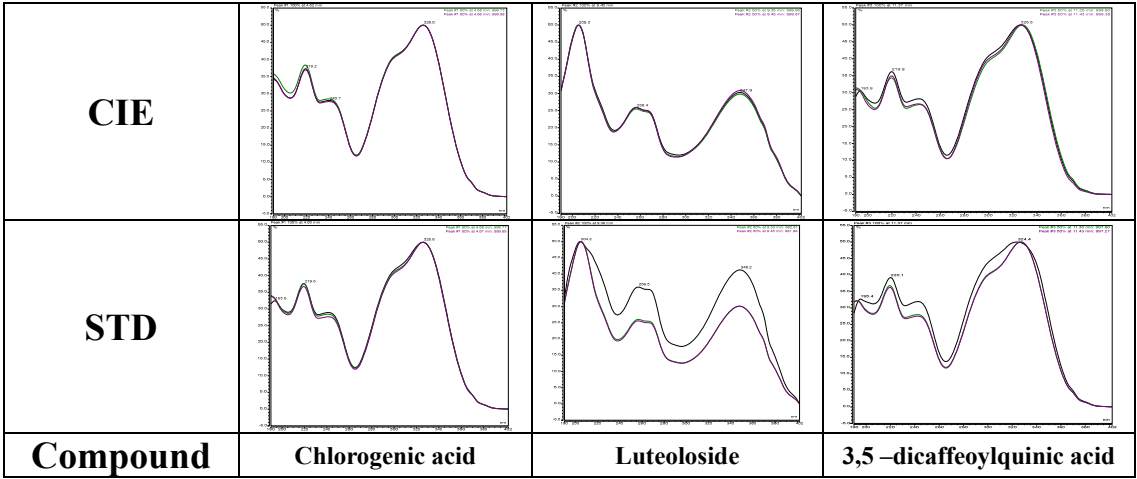

Supplement: Supplementary file 1 [file nutrients-13-03690-s001.zip › nutrients-1400264-supplementary.pdf]
